# Evaluation of atherogenic lipoprotein-cholesterol to HDL cholesterol ratio as a prognostic test for ST-segment elevation myocardial infarction

**DOI:** 10.7150/ijms.44801

**Published:** 2021-06-04

**Authors:** Jia-Yong Li, Wen-Jun Xu, Zhe Zhou, Ru-lin Zhang, Ting Sun, Hao Xu, Jun Wu

**Affiliations:** 1 Department of Laboratory Medicine, Shanghai General Hospital, Shanghai Jiao Tong University School of Medicine, Shanghai, China.; 2 Clinical Laboratory Medicine Center, Shanghai Children's Hospital, Shanghai, China.; 3 Department of Cardiology, Shanghai Ninth people's Hospital, Shanghai Jiao Tong University School of Medicine, Shanghai, China.; 4 Department of Cardiology, Shanghai General Hospital, Shanghai Jiao Tong University School of Medicine, Shanghai, China.; 5 Department of Laboratory Medicine, Shanghai General Hospital Jiading Branch, Shanghai, China.

**Keywords:** remnant lipoprotein cholesterol, atherogenic lipoprotein, STEMI

## Abstract

**Background:** The detectable component of triglyceride-rich lipoproteins (TGRLs), remnant lipoprotein cholesterol (RLP-c), has been proven being correlated with the progression of atherosclerosis and myocardial infarction. However, when taken as a risk predictor, the prognostic and diagnostic potential of RLP-c remains controversial in studies. In this study, we evaluated the hypothesis that atherogenic lipoprotein-cholesterol (AL-c), representing the sum of RLP-c and the sd-LDL-c, to the HDL-c ratio, could represent a better predictive indicator than RLP-c alone in ST-segment elevation myocardial infarction (STEMI).

**Methods:** The 316 consecutive patients suffering from persistent chest discomfort admitted to the Shanghai General Hospital between January 2018 and June 2018 were enrolled. 149 STEMI patients (62% men, mean age 69.6 ± 13.3 years) were included as the study cohort. The AL-c/HDL-c ratio was calculated on admission in a cohort of electrocardiogram-confirmed STEMI patients and compared to other lipid profiles as a predictive indicator.

**Results:** The AL-c/HDL-c ratio was significantly increased in STEMI patients compared with apparently healthy adults (0.93; IQR [0.71-1.18] *vs* 0.70; IQR [0.45-1.04]; *p* < 0.001). Gender dependency existed, and the male and female patients had median AL-c/HDL-c ratios of 1.01 and 0.79, respectively (*p* < 0.001). Compared to RLP-c, the AL-c/HDL-c ratio had a better prognostic value to predict STEMI risk in both sexes (AUC of 0.672 with a sensitivity of 0.794 in males and 0.613 with a sensitivity of 0.684 in females).

**Conclusions:** The AL-c/HDL-c ratio could represent a convenient and sensitive biomarker for screening and predicting STEMI risk.

## Introduction

Myocardial infarction (MI) is one of the leading causes of cardiovascular mortality. The identification of patients at high risk of MI is crucial not only for prevention but also for intervention. STEMI is one type of MI resulted from the complete obstruction of a coronary artery, its onset is significantly associated with dyslipidemia 1,2; therefore, the current protocol to assess the risk of atherosclerotic cardiovascular diseases (CVD), such as MI, involves the quantification of atherogenic lipoproteins 3, including low-density lipoprotein cholesterol (LDL-c), high-density lipoprotein cholesterol (HDL-c) and triglycerides (TGs). LDL-c is indisputably the principal indicator for the assessment of the risk of CVD and the management of dyslipidemia. However, residual risks remain after achieving clinically recommended target levels of LDL-c, indicating that additional biomarkers beyond traditional risk factors are needed 3-5.

Patients at increased risk of CVD are frequently associated with an atherogenic lipoprotein phenotype characterized by elevated levels of both TGRLs and sd-LDL and low concentrations of HDL-c 6. Both HDL-c and sd-LDL are strongly associated with CVD 7,8, but it is controversial whether these factors could serve as independent MI risk predictors 1,9-12. Emerging studies have indicated that TGRLs are significantly associated with CVD 4,13,14; however, the difficulties in measuring TGRLs due to their heterogeneity (consisting of intestinal chylomicrons and hepatic very-low-density lipoproteins and intermediate-density lipoproteins) limit their clinical applications 6,15.

Remnant lipoproteins (RLPs) are metabolic intermediates of TGRLs in the process of conversion into smaller and denser particles by lipoprotein lipase or hepatic lipase 6. The resulting RLPs contain fewer TGs, phospholipids and apolipoprotein-C but more esterified cholesterol and apolipoprotein-E, and these RLPs are believed to be more atherogenic than their larger precursors 6,15. Remnant cholesterol (RLP-c) refers to the cholesterol content of RLPs. Accumulating evidence has demonstrated that increased production and delayed catabolism of TGRLs lead to elevated levels of remnant cholesterol, which is strongly associated with the progression of atherosclerosis 13, increased risk of ischemic heart disease and MI 16-19, residual cardiovascular risk 4,13,14 and total death 19,20. Guidelines 8,21 recommended calculation of non-HDL cholesterol as a measure of RLPs; however, the results would be complicated or poorly interpreted given that the calculated non-HDL cholesterol level contains fewer atherogenic particles 3, and the same situation exists when using TG as an alternative measure 17,22.

Methodologies have been established to detect remnant cholesterols, including the immunoseparation method 23, enzymatic cycling method 24 and homogenous methods 25,26. Studies on TGRLs utilizing these methods have been reported, but controversial results have been achieved regarding remnant cholesterol as an independent risk factor for atherosclerotic cardiovascular diseases 8,27. A massive MI can cause acute heart failure or cardiogenic shock, leading to sudden death in certain scenarios. The identification of high-risk MI would help relieving fatal consequences. Though electrocardiogram (ECG) and troponin are usually enough to diagnose MI, few tools in clinical could identify high-risk MI at early stage. Therefore, innovative indicators are in urgent need to predict the incidence of MI. In the present study, remnant cholesterol levels were measured using a commercial kit developed based on the method reported by Miyauchi and colleagues 25, and we tested the hypothesis that the composite variable, atherogenic lipoprotein-cholesterol (AL-c, the sum of RLP-c and the sd-LDL-c) to HDL-c ratio, which is significantly associated with an atherogenic lipoprotein phenotype concerning these three lipoproteins, represents a better predictive indicator than RLP-c alone in MI.

## Methods

### Baseline data

To establish the baseline characteristics for the AL-c/HDL-c ratio, 295 males and 306 females aged 18 and older were enrolled and analyzed. The subjects were either normolipidemic or hyperlipidemic but were free of symptoms and signs of cardiovascular disease. The study protocol was approved by the Shanghai General Hospital Ethics Committee.

### Study population

A total of 316 consecutive patients suffering from persistent chest discomfort admitted to the Shanghai General Hospital between January 2018 and June 2018 were enrolled, and all underwent 12-lead ECG 24 hours after administration. One hundred forty-nine patients with a final diagnosis of nonfatal MI were included as the study cohort, and all were diagnosed with first STEMI afterwards. STEMI was defined by the presence of symptoms of myocardial ischemia associated with new electrocardiographic abnormalities and subsequent elevation of cardiac biomarkers, such as troponin I, above the 99th percentile of a normal reference population 28. ST-segment elevation measured at the J-point should be found in at least two contiguous leads and be ≥2.5 mm in men < 40 years, ≥ 2 mm in men ≥ 40 years, or ≥ 1.5 mm in women in leads V2-V3 and/or ≥ 1 mm in the other leads [in the absence of left ventricular hypertrophy or left bundle branch block]. Similarly, ST-segment depression in leads V1-V3 suggests myocardial ischemia, especially when the terminal T-wave is positive (ST-segment elevation equivalent) and confirmed by concomitant ST-segment elevation ≥ 0.5 mm recorded in leads V7-V9.

### Laboratory examinations

Nonfasting serum was stored at 4 °C and was used for lipid profile assays within 12 hours after sampling. Total cholesterol (TC), TG, LDL-c, HDL-c, and sd-LDL-c were measured utilizing commercial kits using enzymatic methods on an automatic biochemical analyzer (Siemens ADVIA 2400 Chemistry System). The remnant cholesterol was determined using a commercial enzymatic assay (Shanghai Runho Biotech Ltd., China). The assay principle could be explained by the fact that surfactants cooperating with phospholipase D exhibited favorable selectivity towards remnant lipoproteins, making their component cholesterol available for enzymatic assays. The assay was validated to be equivalent to the RemL-C Kit (Kyowa Medex Co., Ltd., Japan) with a correlation coefficient of 0.98 and an interassay CV of 1.8% in our laboratory. The measurement of cardiac troponin I (cTnI), creatine kinase (CK-MB), and myoglobin (MYO) was performed using commercial chemiluminescence immunoassays on an automatic immunoassay analyzer (Beckman Coulter Access 2 Immunoassay System). Non-HDL-c was calculated as TC minus HDL-c, and the AL-c/HDL-c ratio was calculated as the sum of sd-LDL-c and RLP-c divided by HDL-c. Other data were collected by reviewing the medical records of patients.

### Statistical analysis

All statistical analyses were performed using SPSS 22.0, MedCalc 11.4 and Prism 7 for Windows. Continuous variables are presented as the mean ± standard deviation or median with interquartile range (IQR) and were compared with Student's t-test or by Mann-Whitney U test (if not normally distributed). Categorical variables were expressed as rates or proportions, and comparisons were performed using the chi square test. The Kolmogorov-Smirnov test was used to test the normality of variables. A receiver operating characteristic (ROC) curve was applied to determine the optimal cutoff value of the AL-c/HDL-c ratio in the prediction of STEMI (a minimum specificity of 50% was required for application in practice). Univariate and multivariate logistic regression analysis were performed to identify these risk factors of STEMI. A *P*-value < 0.05 was considered statistically significant.

## Results

### Baseline characteristics and the AL-c/HDL-c ratio of study subjects

The median age of 601 apparently healthy adults was 69.0 years (Table 1). The median TC, TG, LDL-c, HDL-c, sd-LDL-c, RLP-c and non-HDL-c were 4.35, 1.31, 1.08, 2.32, 0.49, 0.24, and 3.20 mmol/L, respectively. No differences in terms of age, TGs, LDL-c, sd-LDL-c or RLP-c were noted between the two sexes. TC and HDL-c levels were higher in females compared with males, showing statistically significant differences. No significant differences in age or other lipid biomarkers were noted between males and females. The median, 25th and 75th percentile AL-c/HDL-c ratios were 0.70, 0.45 and 1.04, respectively, demonstrating a statistically significant difference between the two sexes with medians of 0.74 in males and 0.63 in females. Spearman correlations were utilized to analyze differences between the AL-C/HDL-c ratio and other lipid biomarkers, indicating that the AL-c/HDL-c ratio was positively correlated with the levels of TC, TG, LDL-c, sd-LDL-c, RLP-c and non-HDL-c. In addition, the AL-c/HDL-c ratio was negatively correlated with age and the levels of HDL-c.

### Biomarkers in apparently healthy participants and patients with STEMI

**Figure 1:**
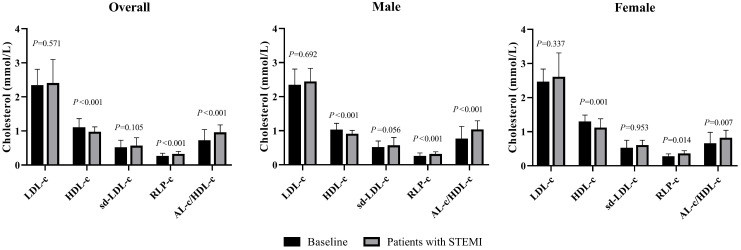
Comparisons of LDL-c, HDL-c, sd-LDL-c, RLP-c and AL-c/HDL-c ratio between patients with STEMI and baseline in male, female and overall study subjects. STEMI: ST-segment elevation myocardial infarction. *P*<0.01 stands for a significant difference between baseline and patients with STEMI.

The average age of 149 subjects with STEMI was 69.6 ± 13.3 years, including 92 males and 57 females. The median cTnI, CK-MB and MYO levels were 0.24, 3.3 and 68.0 ng/mL, respectively, with no statistically significant differences between sexes (Table 2). The female patients were older than the male patients, and the average TC and HDL-c levels were significantly increased. Female patients had a median AL-c/HDL-c ratio of 0.79, which was significantly lower than that of male patients, with a median AL-c/HDL-c ratio of 1.01. No significant differences in other biomarkers were noted between the two sexes. Compared with apparently healthy participants, the overall patients had significantly lower TC and HDL-c levels, higher TG and RLP-c levels, and a higher AL-c/HDL-c ratio with a median of 0.93. Sd-LDL-c and non-HDL-c were slightly increased in patients with STEMI but failed to show statistical significance. Separately, TC, TG, HDL-c and RLP-c levels and the AL-c/HDL-c ratio in male patients with STEMI showed statistically significant differences compared to apparently healthy male participants. In contrast, female patients with STEMI had significantly lower HDL-c levels, higher RLP-c levels, and an increased AL-c/HDL-c ratio compared to baseline characteristics (Table 2 and Fig. 1). Neither sd-LDL-c nor non-HDL-c showed significant differences between patients with STEMI and baseline in the two sexes.

### ROC analysis of AL-c/HDL-c ratio

**Figure 2:**
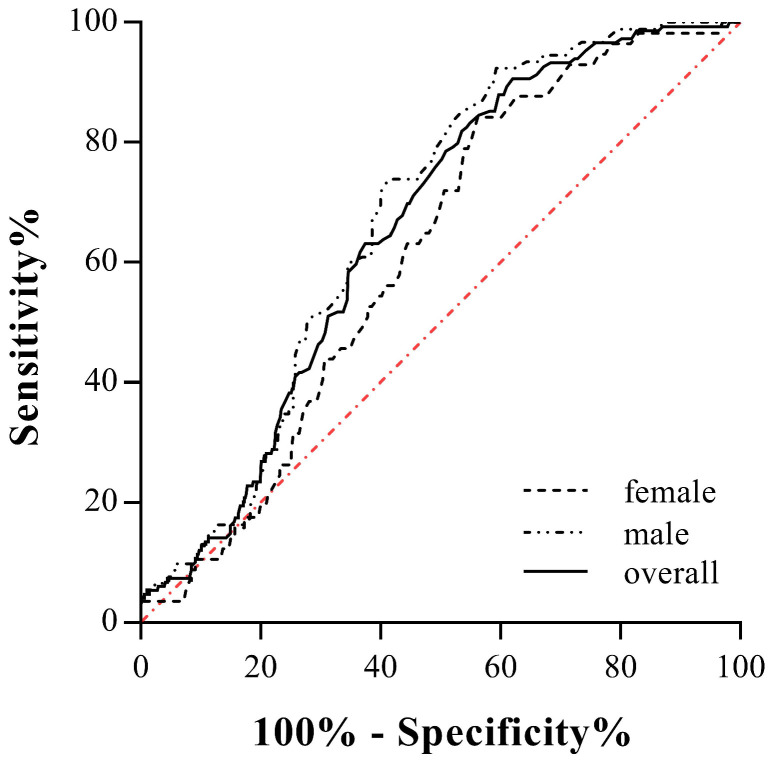
Receiver-operating characteristic (ROC) curves for AL-c/HDL-c ratio in predicting for STEMI in male, female and two genders combined.

The cut-off points of TC, TG, HDL-c, LDL-c and non-LDL-c were 6.20 mmol/L, 2.30 mmol/L, 1.00 mmol/L, 4.10 mmol/L and 4.90 mmol/L, respectively, according to <The Guidelines on Prevention and Treatment of Blood Lipid Abnormality in Chinese Adults> published in 2016. The cut-off values of sd-LDL-c and RLP-c were set at 1.25 mmol/L and 0.31 mmol/L referring to the manufacturer's manual. The area under the ROC curve was 0.672 (95% CI 0.617-0.727, *P*<0.001) for male candidates and 0.613 (95% CI 0.546-0.681, *P*=0.007) for females. The area under the ROC curve was 0.655 (95%CI 0.620-0.689,* P*<0.001) when two genders were calculated together (Fig. 2). For a more clinically useful prediction model, the optimal AL-c/HDL-c cut-off value for predicting STEMI was set at 0.74 with a sensitivity of 79.4% and a specificity of 50.5% for the prediction of STEMI in males, 0.63 with a sensitivity of 68.4% and a specificity of 50.3% in females, and 0.71 with a sensitivity of 74.5% and a specificity of 51.9% for two genders combined. Compared with HDL-c and RLP-c, the AL-c/HDL-c ratio had an equivalent or better AUC and moderate sensitivity but relatively lower specificity. Additionally, sex-based variations showed that the AL-c/HDL-c ratio had better diagnostic potential in males (Table 3).

### Univariate and multivariate regression analysis for the detection of independent predictors of STEMI

**Figure 3:**
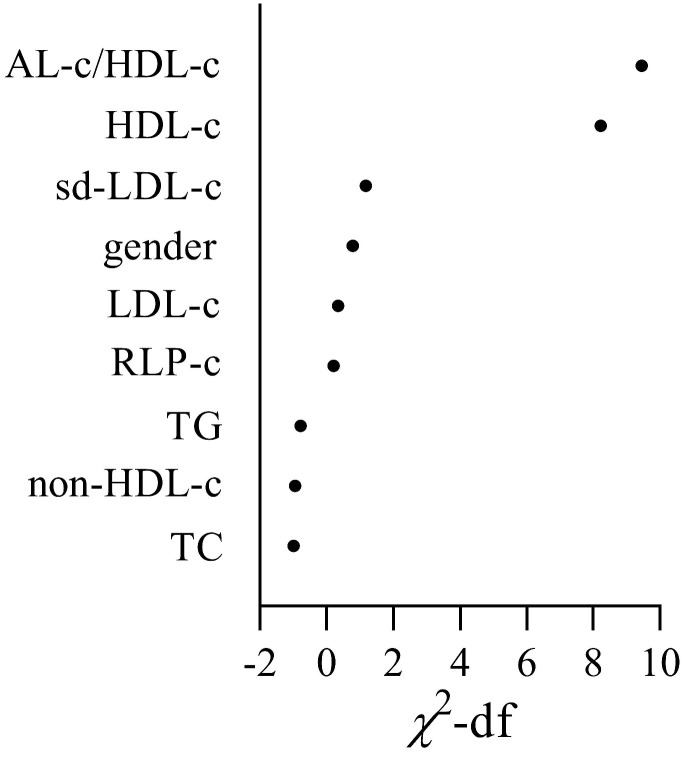
The relative importance of screened risk factors for the prediction of STEMI.

Univariate and multivariate regression analysis was performed to identify the risk factors associated with STEMI (Table 4). The results showed that gender, HDL-c, RLP-c and AL-c/HDL-c ratio were significantly associated with the occurrence of STEMI in univariate regression analyses. Only HDL-c and AL-c/HDL-c ratio was determined independent risk factors associated with STEMI with an odds ratio (OR) of 1.913 (95%CI 1.259-2.908, *P*=0.002) and 2.231 (95%CI 1.372-3.630, *P* = 0.001) respectively, both in univariate and multivariate regression analysis (Table 4). The importance of each screened risk factor in the full model was estimated by the partial chi-square statistic minus the predicted degrees of freedom (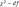, Fig. 3). The AL-c/HDL-c ratio was the most critical risk factor, followed by the HDL-c concentrations.

## Discussion

Proteins, such as myoglobin, cardiac troponin and CK-MB, were released from necrotic cardiomyocytes when MI occurred, and all of these proteins are known diagnostic biomarkers for MI. Although studies have suggested that higher concentrations of cardiac troponin are independently associated with an increased risk of cardiovascular events in patients with acute coronary syndromes 29, cardiac troponin is mostly used as a diagnostic criterion rather than earlier predictor in clinical practice. Moreover, elevation of cardiac troponin is also occurred in myocarditis, pericarditis, heart failure and other diseases 30. More performant and economic biomarkers are urgently needed to be integrated into routine clinical implications as predictive biomarkers for MI.

Disorders of TGRL metabolism are crucial in the pathogenesis of CVD, and the process also involves other lipoprotein changes, including reduction of HDL-c and elevation of sd-LDL-c, with or without elevated LDL-c levels. The metabolic interrelations among TGRLs, HDL-c and sd-LDL-c make it difficult to determine the independent contributions of these factors to CVD risks 31, and previous studies on TGRLs have reported controversial conclusions regarding their use as an independent CVD risk predictor. Nonfasting TG levels are an independent predictor of MI in women, and increased nonfasting TG levels may indicate the presence of increased levels of atherogenic RLPs 19. The mechanism could be explained by the fact that denser RLPs can enter the arterial intima and be trapped in the arterial wall, thus causing atherosclerosis and CVD onset. The results of our study were summarized as follows: 1) The baseline AL-c/HDL-c ratio in apparently healthy adults differed between sexes with medians of 0.74 in males and 0.63 in females. 2) The AL-C/HDL-c ratio was positively correlated with TC, TG, LDL-c, sd-LDL-c, RLP-c and non-HDL-c levels and negatively correlated with age and HDL-c levels. 3) Overall, patients with STEMI had a significantly higher AL-c/HDL-c ratio, and sex dependency also existed. Specifically, male and female patients had median AL-c/HDL-c ratios of 1.01 and 0.79, respectively, both of which were increased compared with their healthy counterparts. 4) Traditional biomarkers, such as LDL-c, sd-LDL-c and non-HDL-c, failed to show significant differences between patients with STEMI and baseline in the two sexes. 5) Cut-off values of 0.74, 0.63 and 0.71 were determined to be the optimal AL-c/HDL-c value for predicting STEMI in males, females and two genders combined, respectively. Compared with RLP-c, the AL-c/HDL-c ratio had the better sensitivity and specificity for the prediction of STEMI than RLP-c alone and even traditional biomarkers, such as LDL-c and HDL-c. 6) Univariate and multivariate regression analysis showed that HDL-c and AL-c/HDL-c ratio were independent risk predictors associated with STEMI. All these data suggested that the AL-c/HDL-c ratio could help predicting the upcoming risk of cardiovascular events given that acute MI is the consequent outcome of the accumulation of atherogenic lipoproteins.

**Figure 4:**
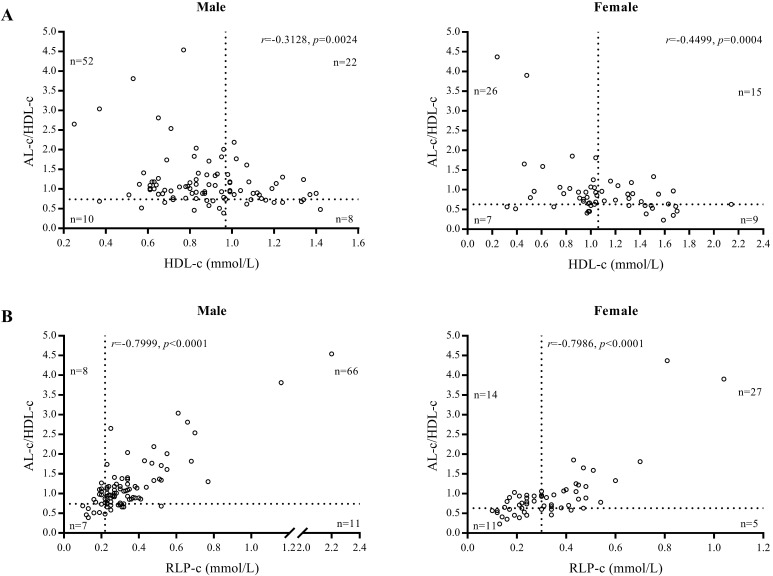
Gender-based scatter plot of AL-c/HDL-c ratio vs. HDL-c of patients with STEMI (A) and AL-c/HDL-c ratio vs. RLP-c of patients with STEMI (B).

We found AL-c/HDL-c was associated with STEMI and was an independent marker for predicting the risk of STEMI in both men and women with high sensitivity. This conclusion was supported by our findings demonstrating that 74 of 92 male and 41 of 57 female patients had elevated AL-c/HDL-c ratios (cut-off values of 0.74 and 0.63), whereas only 30 male and 24 female patients were validated as positive when using HDL-c as a diagnostic biomarker (cut-off values of 1.06 and 0.97 mmol/L) (Fig. 4A). Compared with RLP-c, the AL-c/HDL-c ratio confirmed that 80.4% of male and 71.9% of female patients were positive, whereas 15 male patients (16.3%) and 25 female patients (43.9%) had a false-negative level of RLP-c (cut-off values of 0.22 and 0.30 mmol/L) (Fig. 4B). In our study, the diagnostic performance of AL-c/HDL-c was shown prior in males (Table 4). The sex variation in the AL-c/HDL-c ratio was inherited from HDL-c level, which may indicate the different pathophysiology of CVD and heart failure between sexes 29. Notably, the difference remained doubtful since the scale of female candidates was relatively small than male, further research was needed to validated the sex variation.

Compared to HDL-c and RLP-c, the AL-c/HDL-c ratio had high diagnostic sensitivity for STEMI (Table 3). This characteristic makes it a promising screening test for unidentified patients with STEMI risks. Considering the severity and high mortality of STEMI, a higher sensitivity and relatively low specificity (<50%) should be considered given that the early prognosis of patients with STEMI is crucial for more extensive investigations and more aggressive treatment. We believe that our results suggest that AL-c/HDL-c could be particularly useful in identifying high-risk acute MI patients who are free of symptoms and have under controlled LDL-c levels because both LDL-c and sd-LDL-c failed to be independent risk predictors in this study. When validated with an elevated AL-c/HDL-c ratio, patients should be monitored with intensive care and more frequent follow-up.

## Limitations of the study

Our study limitations include that the present study was a single-center study with a relatively small population. Nonfasting serum was collected and measured in this study, which could be a potential disturbance leading to inaccurate measurements of LDL-c and other lipids. Additionally, the assay used to determine remnant cholesterols differed from other studies, which may cause different results given the heterogeneities of RLPs. Importantly, the present work is exploratory based on this hypothesis, and further studies are required in larger cohorts to confirm the predictive value of AL-c/HDL-c compared to other biomarkers.

## Conclusion

The results of the present work suggest that AL-c/HDL-c could represent a convenient and sensitive biomarker for screening and predicting high-risk of STEMI. Abnormally elevated values suggest a potential possibility of acute STEMI, and interventions should be followed for precaution in these high-risk patients.

## Figures and Tables

**Table 1:** Baseline characteristics of 601apparently healthy participants based on gender

Variables	Overall	Correlation coefficients	Male	Female	*P* value
**Demographic characteristics**					
n, (%)	601 (100%)	-	295 (49.1%)	306 (50.9%)	-
Age, years	69.0 (59.0, 83.0)	-0.150	67.0 (59.0, 83.0)	72.0 (61.0, 83.0)	0.065
**Biochemical indicators**					
TC, mmol/L	4.35 (3.74, 5.18)	0.306	4.29 ± 1.07	4.67 ± 1.10	<0.001
TG, mmol/L	1.31 (0.87, 1.88)	0.776	1.30 (0.92, 1.83)	1.32 (0.83, 1.90)	0.811
HDL-c, mmol/L	1.08 (0.91, 1.36)	-0.551	1.00 (0.84, 1.22)	1.27 ± 0.39	<0.001
LDL-c, mmol/L	2.32 (1.89, 2.81)	0.438	2.32 (1.86, 2.72)	2.44 ± 0.74	0.132
sd-LDL-c, mmol/L	0.49 (0.36, 0.73)	0.784	0.49 (0.38, 0.70)	0.50 (0.36, 0.75)	0.865
RLP-c, mmol/L	0.24 (0.16, 0.35)	0.773	0.23 (0.15, 0.35)	0.25 (0.16, 0.35)	0.365
Non-HDL-c, mmol/L	3.20 (2.62, 3.93)	0.535	3.24 ± 1.01	3.39 ± 1.00	0.057
AL-c/HDL-c ratio	0.70 (0.45, 1.04)	-	0.74 (0.49, 1.13)	0.63 (0.42, 0.98)	0.002

**Table 2:** General characteristics of study subjects with STEMI

Variables	Overall	*P* value^a^	Male	*P* value^a^	Female	*P* value^a^	*P* value^b^
**Demographic characteristics**							
n, (%)	149 (100%)	-	92 (61.7%)	-	57 (38.3%)	-	-
Age, years	69.6 ± 13.3	0.691	65.2 ± 13.4	0.032	76.7 ± 9.7	0.011	<0.001
**Biochemical indicators**							
TC, mmol/L	4.23 ± 1.25	0.013	4.03 ± 1.10	0.044	4.56 ± 1.40	0.592	0.010
TG, mmol/L	1.42 (1.07, 1.95)	0.015	1.41 (1.13, 1.90)	0.030	1.55 ± 0.67	0.209	0.650
HDL-c, mmol/L	0.95 (0.75, 1.12)	<0.001	0.88 ± 0.24	<0.001	1.09 ± 0.39	0.001	<0.001
LDL-c, mmol/L	2.38 (1.78, 3.10)	0.571	2.42 ± 0.91	0.692	2.58 ± 1.01	0.337	0.306
sd-LDL-c, mmol/L	0.54 (0.41, 0.80)	0.105	0.64 ± 0.31	0.056	0.58 ± 0.29	0.953	0.235
RLP-c, mmol/L	0.30 (0.23, 0.40)	<0.001	0.29 (0.23, 0.38)	<0.001	0.33 ± 0.18	0.014	0.992
Non-HDL-c, mmol/L	3.27 ± 1.07	0.571	3.15 ± 0.98	0.451	3.47 ± 1.17	0.601	0.071
AL-c/HDL-c ratio	0.93 (0.71, 1.18)	<0.001	1.01 (0.78, 1.29)	<0.001	0.79 (0.60, 1.04)	0.007	<0.001
cTnI, ng/mL	0.24 (0.10, 0.97)	-	0.24 (0.10, 0.97)	-	0.18 (0.07, 0.89)	-	0.286
CK-MB, ng/mL	3.3 (1.5, 7.0)	-	3.3 (1.5, 7.0)	-	3.1 (1.6, 6.2)	-	0.318
MYO, ng/mL	68.0 (36.2, 144.5)	-	68.0 (36.2, 144.5)	-	71.4 (42.8, 141.3)	-	0.250

^a^ *P* value compared with Control group; ^b^ *P* value compared between two genders.

**Table 3:** Comparisons of diagnostic sensitivity, specificity, and accuracy of various biomarkers

Biomarkers	HDL-c	sd-LDL-c	RLP-c	AL-c/HDL-c
**Male**				
Cut-off value	0.97 mmol/L	0.50 mmol/L	0.22 mmol/L	0.74
Area under curve	0.668 (0.605, 0.730)	0.566 (0.034, 0.056)	0.630 (0.573, 0.688)	0.672 (0.617, 0.727)
Sensitivity	0.674 (0.568, 0.768)	0.652 (0.546, 0.749)	0.837 (0.745, 0.906)	0.794 (0.696, 0.871)
Specificity	0.583 (0.524, 0.640)	0.506 (0.447, 0.564)	0.468 (0.410, 0.527)	0.505 (0.447, 0.564)
*P* value	<0.001	0.056	<0.001	<0.001
**Female**				
Cut-off value	1.06 mmol/L	0.28 mmol/L	0.30 mmol/L	0.63
Area under curve	0.618 (0.537, 0.699)	0.502 (0.420, 0.585)	0.603 (0.527, 0.679)	0.613 (0.546, 0.681)
Sensitivity	0.579 (0.441, 0.670)	0.175 (0.087, 0.299)	0.561 (0.424, 0.693)	0.684 (0.548, 0.801)
Specificity	0.670 (0.614, 0.722)	0.918 (0.882, 0.946)	0.628 (0.571, 0.682)	0.503 (0.446, 0.561)
*P* value	0.005	0.953	0.014	0.007
**Overall**				
Cut-off value	1.04 mmol/L	0.47 mmol/L	0.21 mmol/L	0.71
Area under curve	0.663 (0.628, 0.697)	0.543 (0.506, 0.579)	0.617 (0.581, 0.651)	0.655 (0.620, 0.689)
Sensitivity	0.698 (0.617, 0.770)	0.631 (0.548, 0.708)	0.799 (0.725, 0.860)	0.745 (0.667, 0.813)
Specificity	0.556 (0.515, 0.596)	0.476 (0.435, 0.517)	0.438 (0.398, 0.478)	0.519 (0.478, 0.560)
*P* value	<0.001	0.099	<0.001	<0.001

**Table 4:** Univariate and multivariate regression analysis for the detection of independent relationship with the occurrence of STEMI

Variables	Univariate analysis OR (95% CI)		Multivariate analysis OR (95% CI)	
	OR (95%CI)	*P*	OR (95%CI)	*P*
Gender	0.597 (0.414-0.862)	0.006	0.766 (0.517-1.133)	0.182
TC	1.057 (0.545-2.049)	0.870	0.925 (0.217-3.931)	0.915
TG	1.622 (0.312-8.442)	0.566	1.553 (0.252-9.576)	0.636
HDL-c	2.697 (1.864-3.902)	<0.001	1.913 (1.259-2.908)	0.002
LDL-c	1.648 (0.711-3.819)	0.244	2.108 (0.599-7.422)	0.246
non-LDL-c	1.168 (0.627-2.178)	0.625	0.847 (0.227-3.156)	0.804
sd-LDL-c	0.890 (0.405-1.958)	0.773	0.523 (0.221-1.238)	0.140
RLP-c	1.802 (1.249-2.601)	0.002	1.282 (0.825-1.992)	0.270
AL-c/HDL-c	3.154 (2.110-4.712)	<0.001	2.231 (1.372-3.630)	0.001
